# Molecular Dynamics of Mesophilic-Like Mutants of a Cold-Adapted Enzyme: Insights into Distal Effects Induced by the Mutations

**DOI:** 10.1371/journal.pone.0024214

**Published:** 2011-09-07

**Authors:** Elena Papaleo, Marco Pasi, Matteo Tiberti, Luca De Gioia

**Affiliations:** Department of Biotechnology and Biosciences, University of Milano-Bicocca, Milan, Italy; King′s College London, United Kingdom

## Abstract

Networks and clusters of intramolecular interactions, as well as their “communication” across the three-dimensional architecture have a prominent role in determining protein stability and function. Special attention has been dedicated to their role in thermal adaptation. In the present contribution, seven previously experimentally characterized mutants of a cold-adapted α-amylase, featuring mesophilic-like behavior, have been investigated by multiple molecular dynamics simulations, essential dynamics and analyses of correlated motions and electrostatic interactions. Our data elucidate the molecular mechanisms underlying the ability of single and multiple mutations to globally modulate dynamic properties of the cold-adapted α-amylase, including both local and complex unpredictable distal effects. Our investigation also shows, in agreement with the experimental data, that the conversion of the cold-adapted enzyme in a warm-adapted variant cannot be completely achieved by the introduction of few mutations, also providing the rationale behind these effects. Moreover, pivotal residues, which are likely to mediate the effects induced by the mutations, have been identified from our analyses, as well as a group of suitable candidates for protein engineering. In fact, a subset of residues here identified (as an isoleucine, or networks of mesophilic-like salt bridges in the proximity of the catalytic site) should be considered, in experimental studies, to get a more efficient modification of the features of the cold-adapted enzyme.

## Introduction

A detailed comprehension of molecular mechanisms that rule the relationship between stability, flexibility, and activity in extremophilic enzymes is of crucial importance both for fundamental and applicative research [1]–[3]. Enzymes isolated from psychrophilic organisms have received particular attention from the scientific community in the last 20 years, thanks to their unique properties in terms of high activity at detrimental temperatures, low thermal stability and unusual specificity, offering a wide spectrum of industrial applications [4], [5].

The increasing number of primary sequences and three-dimensional (3D) structures of enzymes from extremophiles [6]–[8] has provided a suitable background to disclose molecular determinants of their structural stability. It is well established that psychrophilic enzymes use different adaptation strategies [9], [10], with each protein family adopting its own structural strategy [9], [11].

The molecular determinants and the exact relationships between activity, stability and flexibility in cold-adapted enzymes are still a matter of debate. In fact, the intrinsic thermolability and increased low temperature activity of psychrophilic enzymes prompt for a direct link between activity and stability [12]. Otherwise, it has been suggested that thermolability may be associated to a lack of evolutionary pressure for stable enzymes in low temperature habitats [13], [14]. The existence of non-canonical cold-adapted enzymes, featuring both unusual thermal stability and high catalytic efficiency at low temperatures [15], [16], along with the capability to uncouple activity and stability in *in vitro* evolution studies [17], make the definition of activity-stability-flexibility trade-off even more difficult.

**Figure 1:**
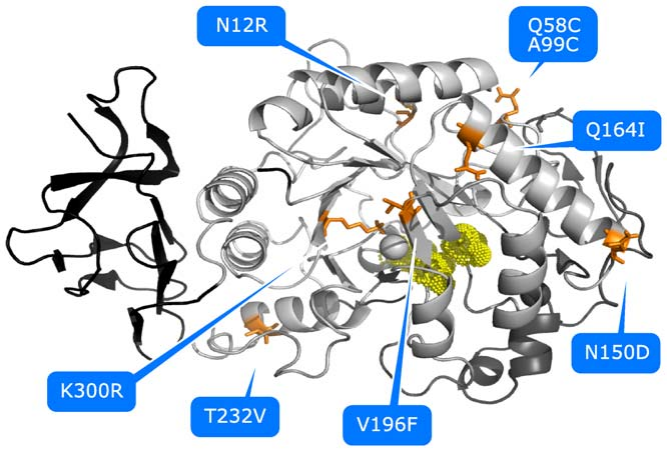
AHA mutants engineered in order to restore interactions typical of the warm-adapted homolog, PPA. Localization of all the mutations included in the simulated AHA mutants, on the 3D structure. Secondary structure elements are indicated as cartoon, white, grey and black cartoons indicate domain A, B and C, respectively. The yellow dots and sticks indicate the localization of the catalytic triad, the spheres Ca2+ and Cl− cofactors and the mutated residues are indicated as orange sticks.

Structural flexibility and rigidity are likely to cooperate, each acting on specific areas of the enzyme structure, nevertheless, they are difficult to quantify for a small and anisotropic material such as a protein molecule [9], [12]. In this context, the current view on the relationships between protein dynamics and function [18]–[22] suggests that protein function is rooted in the free energy landscape [19] and that fluctuations at equilibrium can influence biological functions. In fact, backbone flexibility profiles diverge slowly, being conserved both in protein family and superfamily [20], [23], [24]. However, a recent study has shown that warm- and cold-adapted enzymes belonging to the same family present common dynamics signature related to the same fold, but also specific differences which may reflect temperature adaptation [25]. In fact, the striking correspondence between picosecond dynamics and longer scale conformational changes suggests that the physical origin of functionally important collective motions is the fast time-scale local motions [19] and that differences in the fast fluctuations are encoded by differences in the primary sequence. Moreover, as results of recent advances both in biophysical spectroscopies and molecular dynamics (MD) simulations, it is possible to extract detailed information on coupled motions and networks of communicating residues in the 3D structure and during dynamics, thanks for example to analysis of cross-correlation of atomic fluctuations [26]–[28], or long-range pathway of communicating residues [28]–[31]. The psychrophilic chloride-dependent [32] α-amylase from the Antarctic bacterium *Pseudoalteromonas haloplanktis* (namely AHA) is a paradigm for the study of molecular determinants of enzyme cold-adaptation. In particular, AHA was one of the first psychrophilic enzymes for which the 3D structure was solved [33] (Figure 1), revealing the presence of three distinct structural domains (namely A, B and C). It has been suggested that AHA acquires a low conformational stability and high flexibility through a reduction or weakening of inter- and intra-domain interactions, as well as it features decreased activation enthalpy and a concomitant improvement in k_cat_ if compared to mammalian α-amylases, as pig pancreatic α-amylase (PPA) [34]. AHA site-directed mutagenesis [35]–[37] has been insightful in clarifying the relationships between activity and structural stability. In particular, single and multiple mutants were investigated to restore weak intra-molecular interactions, which are present in the closest mesophilic homolog, PPA. It turns out that, the reestablishment of weak interactions in AHA produces variants with both thermal stability and catalytic efficiency shifted toward “mesophilic-like” values (Figure S1 in Supporting Information S1). The majority of the investigated mutations feature increase in T_m_ (melting temperature) and a decrease in both k_cat_ and K_m_ [35], [37], [38]. The authors speculated that structural stabilization may be related to improved rigidity of the active site: reducing its flexibility, in fact, would increase the activation energy [39], leading to a reduction of k_cat_ values, and would constrain the ground-state of the enzyme-substrate complex to a narrower distribution of conformational states, thus lowering K_m_.

In the present study, seven of the previously characterized AHA mutants [35], [37], [38] featuring the most clear-cut effects on activity and thermal stability (Figure 1, Figure S1 in Supporting Information S1), were investigated by multiple MD simulations in explicit solvent (collecting more than 0.25 µs, overall). The aim of our study is the elucidation of effects induced by the mutations in atomic details with particular attention to long range effects, as well as to identify the determinants of the uncompleted conversion of AHA mutants in mesophilic-like variants. The restored weak interactions are capable of modifying the AHA dynamics in the direction of the warm-adapted enzyme, inducing a complex array of long range effects. Our results also point out a subset of critical residues for PPA and AHA dynamics and structure, not previously identified, which can be a suitable test case for AHA protein engineering in a mesophilic-like direction.

## Results

### Local effects induced by the mutations in the AHA mutant variants

A detailed structural characterization of the AHA mutant variants investigated by D'Amico et al. [35], [37] was not previously carried out and, advantaged by the available dynamic framework, we have initially evaluated whether the mutations have effectively restored the targeted weak interactions. A description of local effects induced by the substitutions is also provided, by monitoring the persistence of residues in the surroundings of the mutations. The interactions hypothesized by D'Amico and coworkers were successfully restored (Figure S5–S9 in Supporting Information S1), although our dynamic analysis points out more complex effects and interaction networks present in PPA, which have to be considered and which can also explain the weak overall effects induced by some mutations on the kinetic and thermodynamic properties of AHA. The networks which are not restored in the AHA mutants, due to several amino acidic substitutions with respect to PPA, interest in particular electrostatic interactions as salt bridges and aromatic clusters.

*N12R* mutation restores the salt bridge between R12^A^ (R20^P^ in PPA), on β1-α1 loop and D15^A^ (D23^P^) on α1 (Figure 1, Figure S2 in Supporting Information S1). In PPA R20^P^ forms a network with both D23^P^ and E369^P^ (which cannot be established in AHA due to P319^A^) and via its aliphatic carbons interact with hydrophobic residues in β8-α8 loop (366–370^P^, Figure S3 in Supporting Information S1), successfully restored in AHA.

*N150D* mutation reestablishes, in AHA, a surface salt bridge between D150^A^, on α3, and K190^A^ on α4 (Figure 1, Figure S3B in Supporting Information S1). However, in PPA the corresponding D173-K213^P^ bridge belongs to a more complex cluster of electrostatic interactions, which extends through domain B, connecting it with the proximity of the catalytic domain, and cannot be established in AHA due to several substitutions (see below).

**Figure 2:**
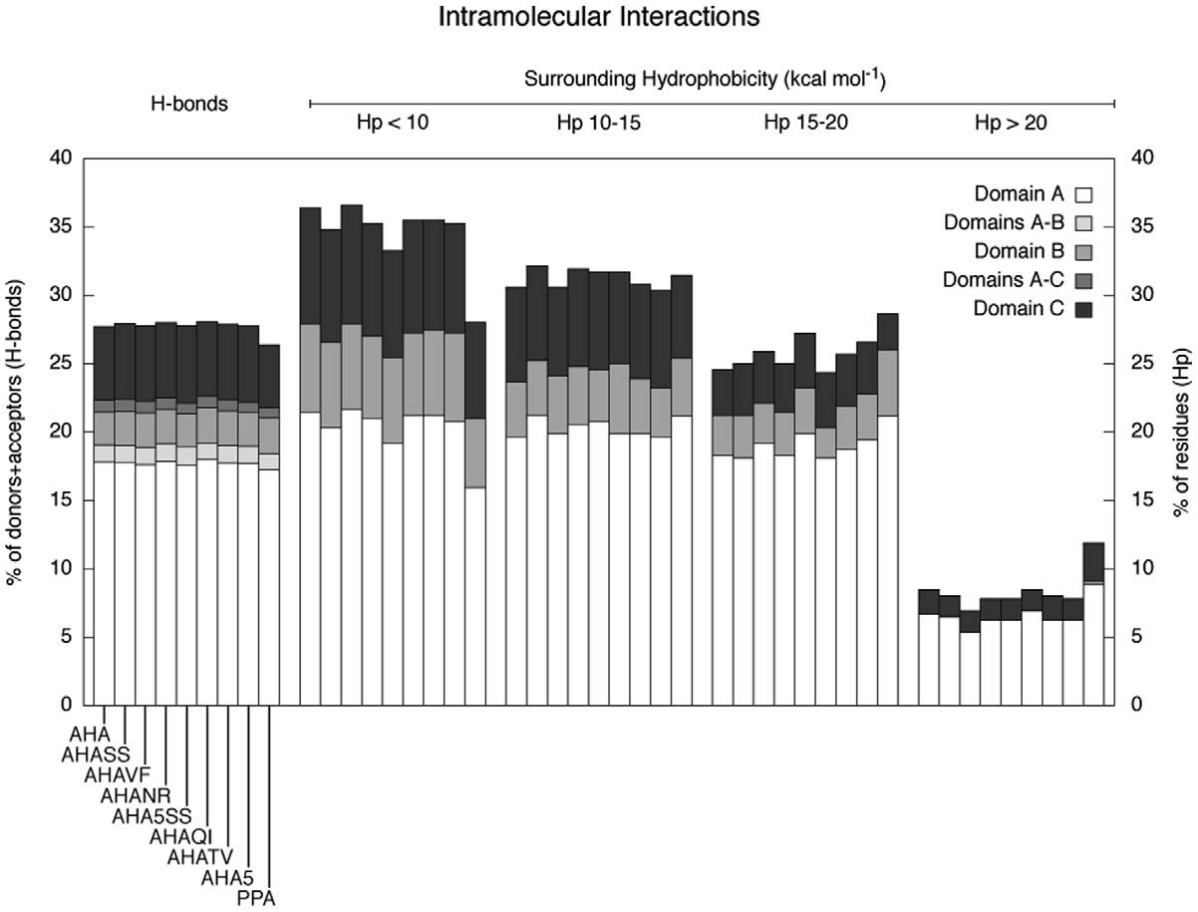
Hydrogen bonds and surrounding hydrophobicity (Hp) are described as histograms representing the percentage of donors+acceptors involved in H-bonds (first set, y axis on the left) or of residues with surrounding hydrophobicity in the indicated range (Hp, all other sets, y axis on the right); each set shows values computed for AHA, PPA and the mutants.

**Figure 3:**
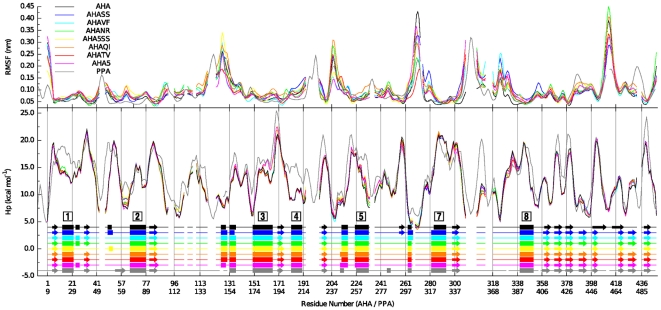
Rmsf in the essential subspace and surrounding hydrophobicity profiles, plotted as a function of the sequences of AHA (labels in the top row) and PPA (labels in the bottom row), and smoothed using window-averaging (window-size = 5 residues). A schematic representation of the most frequently attained secondary structure is shown in the lower plot, where α-helices of the α/β-barrel structure of domain A are labelled according to their order in the primary sequence.

*Q164I* mutation reinforces a hydrophobic cluster including α3 (150–166^A^), β4 (170–174^A^), as well as α3-β4 and α4-β5 (190–200^A^) loops (Figure 1, Figure S3C–D in Supporting Information S1). A clear increase in Hp is observed for AHA mutants which bear Q164I mutation (AHAQI, AHA5SS and AHA5) thanks to both local (α3) and distal (α4-β5) effects, resulting in a global increase of residues with high surrounding hydrophobicity (Hp>20 kcal/mol) (Figure 2 and 3).

*V196F* mutation, in the proximity of Q164I and K300R mutations (Figure 1, Figure S3D and S4 in Supporting Information S1), has been engineered to re-establish two aromatic interactions between F196^A^ (β5), and residues Y82^A^ and F198^A^ (corresponding to Y94^P^ and F291^P^ in PPA), on β3 and β5 respectively (Figure S4 in Supporting Information S1). In PPA, F229^P^ is part of a larger cluster of aromatic and hydrophobic residues, which also interact with the protein N-terminal extremity (residues 1–13^P^); whereas in AHA the N-terminal is 8 residues shorter, thus not allowing the extension of the network (Figure S5A in Supporting Information S1). Moreover, the presence of two arginines (R92^P^ and R252^P^) in the macro-aromatic cluster of PPA, and not in AHA, accounts for a further stabilizing contribution due to cation-PI interactions. AHA mutants bearing V196F (AHAVF, AHA5SS and AHA5) show a local increase in the surrounding hydrophobicity (Figure 3), thanks to the favorable packing induced by mutation, even in absence of the reconstruction of other more complex interactions.

*T232V* mutation strengthens a hydrophobic cluster between α6 and α6-β7, in the proximity of the interface between domains A and C (Figure 1, Figure S5B–C in Supporting Information S1). AHA variants bearing T232V mutation (AHATV, AHA5SS and AHASS) show a higher persistence of hydrophobic residues and a consequent increase in surrounding hydrophobicity (Figure 3), including in particular L238^A^, F244^A^ and W248^A^ (Figure S5C in Supporting Information S1).

*K300R* mutation, in the chloride-binding site and in the proximity of the active site (Figure 1), should provide bi-dentate coordination for the Cl^−^ ion, even if a more complex network of interactions is mediated by R337^P^ in PPA (Figure S5D and S6 in Supporting Information S1). In fact, the mutation is located among three macro-clusters of aromatic residues, conserved in both AHA and PPA (Figure S6 in Supporting Information S1). The K300R mutation has slight effects on the persistence of the aromatic residues in the surrounding of the mutated residues (Figure S5D in Supporting Information S1), while it strengthens a salt bridge with D261 (R337^P^-D297^P^ in PPA). This is in line with the observed role of arginine if compared to lysine in an aromatic context [40], role which is not ascribable to a intrinsic higher cation-PI binding ability of Arg. In fact, the Arg side chain is larger and less water-solvated than the cognate Lys thus, likely to benefit from a better van der Waals interactions with aromatic rings. Moreover, as suggested also by Thornton and colleagues [41], the Arg side chain can still be involved in H-bonds or salt bridges while simultaneously interacting with aromatic rings, whereas Lys typically has to relinquish H-bonds to bind to aromatic residues.

### Effects of the mutation on the structural and dynamic properties of AHA

**Figure 4:**
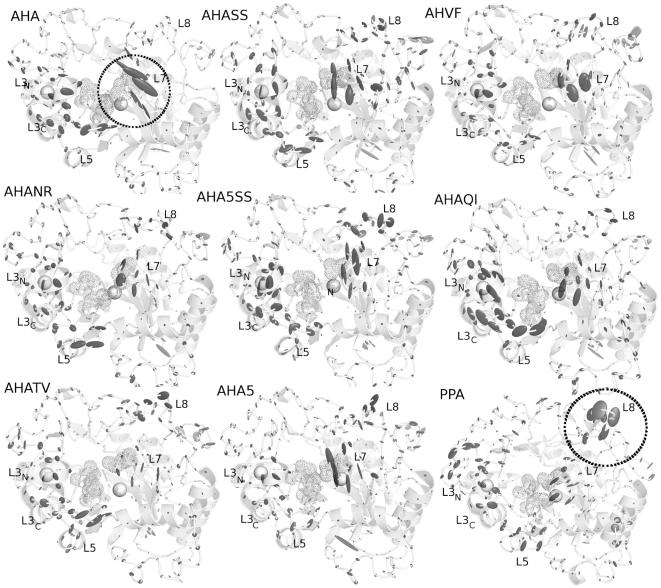
Anisotropic temperature factors computed for fluctuations in the essential subspace. The anisotropic temperature factors are shown as grey ellipsoids centred on the Cα atoms of the average structures from simulations. Grey spheres represent the coordinated ions. The regions of loop 7 and 8 are highlighted by circles in AHA and PPA, respectively.

**Figure 5:**
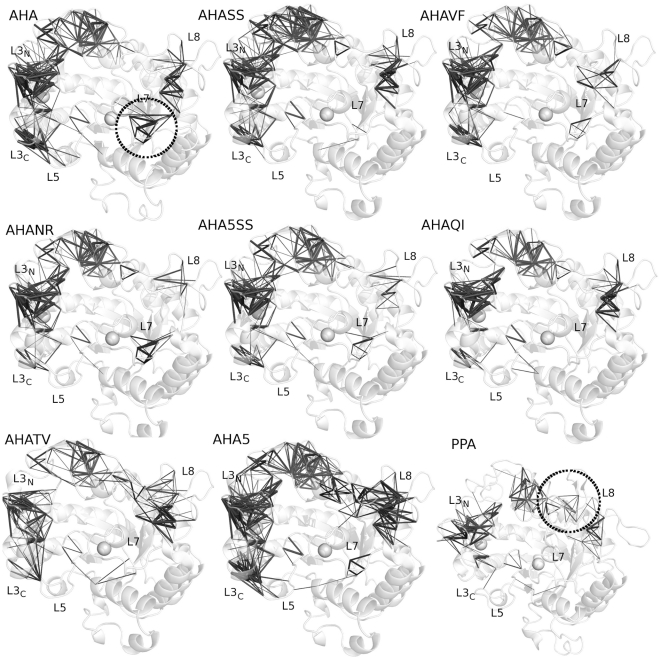
Significant positive correlations between Cα atoms of domains A and B. The positive correlations are represented as grey sticks connecting the Cα atoms of the average structures from simulations. Grey spheres represent the coordinated ions. The regions of loop 7 and 8 are highlighted by circles in AHA and PPA, respectively.

**Figure 6:**
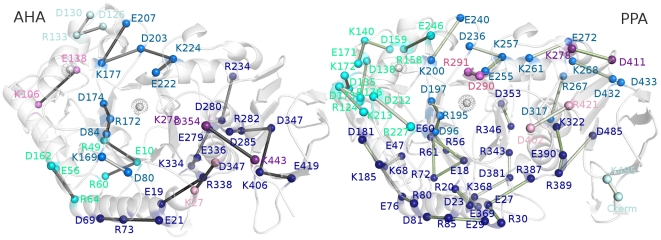
Salt bridges clusters in wild-type AHA and PPA simulations. The salt bridges are mapped on the 3D average structures from simulation as sticks connecting the Cα atoms for AHA and PPA. The white dot and spheres indicate the location of the coordinated ions. The different clusters of spatial proximity of the salt-bridges and their networks are indicated by different colors (blue, lightblue, cyan, palecyan, purple, violet, pink, green, dark green and white for clusters 1,2,3,4,5,6,7,8,9 and 10, respectively). A detailed list of the salt bridges is reported in Table S1 (in Supporting Information S1).

**Figure 7:**
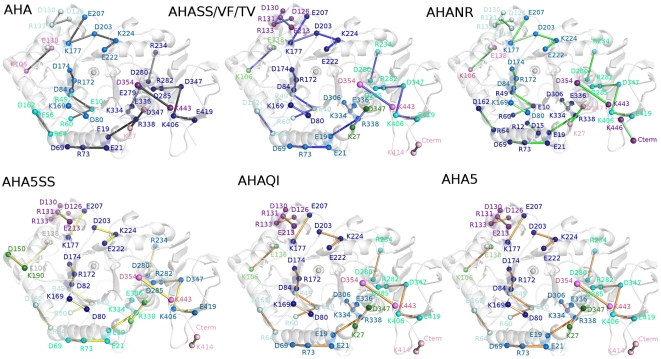
Salt bridge clusters in AHA mutants - part I. The salt bridges are mapped on the 3D average structures from simulation as sticks connecting the Cα atoms for AHASS, AHAVF, AHATV, AHANR, AHAQI, AHA5SS and AHA5. Salt-bridges of wild-type AHA are reported as a reference. (nota: meglio rimandare alla legenda di figura 6?) The white dots and spheres indicate the location of the coordinated ions. The different clusters of spatial proximity of the salt-bridges and their networks are indicated by different colors (blue, lightblue, cyan, palecyan, purple, violet, pink, green, dark green and white for clusters 1,2,3,4,5,6,7,8,9 and 10, respectively). A detailed list of the salt bridges is reported in Table S1 (in Supporting Information S1).

To achieve an overall description of the structural and dynamic properties of the AHA mutants, several properties have been analyzed, from H-bonds and surrounding hydrophobicity (Hp) (Figure 2 and 3), rmsf profiles (Figure 3), the anisotropic temperature factors of the essential subspace (Figure 4), most relevant positive correlated motions (Figure 5), and salt bridge interactions and networks (Figure 6, 7, Figure S7 and Table S1 in Supporting Information S1), along with the clusters of salt-bridges defined by a spatial proximity criterion (Figure 6, 7, Figure S7 and Table S1 in Supporting Information S1).

In fact, it is well-known that the protein stability results from a delicate balance between different weak intramolecular interactions, which in turn also modulate protein dynamics. Electrostatic interactions, and in particular salt-bridges, have been shown to play a crucial role in protein stability [42]–[45], featuring both local and distal variable effects [46], [47].

Salt-bridges are generally highly flexible and cooperatively organized in salt-bridge networks in the protein structure, strongly influencing protein dynamics. Therefore, they are a suitable group of intramolecular interactions which may be used as a reference to identify dynamic intramolecular networks and how they are modified by mutations. In light of these observation, we define not only salt-bridge pairs and networks, according to their persistence during the simulations, but also how they are organized in spatial proximity clusters, as previously applied to other comparative studies on extremophilic enzymes [48]. Our description does not account for calculations of salt-bridge strength and its influence on protein stability [46], [47], [49], which still lacks an accurate definition in extremophilic α-amylases, both since experiments and simulations at different temperatures are still not available and also since most of the residues involved in salt-bridges which differ between AHA and AHA mutants are generally solvent exposed (with a solvent accessibility of their side chains generally higher than 30% during the simulation time) and therefore likely not to be negatively influenced by desolvation penalties.

Globally, few differences, mainly in domain A, are observed in terms of H-bond content and surrounding hydrophobicity (Figure 2 and 3). All mutants show a reduced content of residues with low surrounding hydrophobicity, an increase in residues characterized by higher Hp values, especially the variants which include hydrophobic or aromatic mutations (AHA5SS, AHA5, AHATV, AHAQI, AHAVF) (Figure 2 and 3), as also discussed above.

The patterns of correlated motions and flexibility of AHA and PPA present distinctive features, which have been described in details in a previous publication [50]. The main relevant aspect is related to βα loops of different length and composition in the surroundings of the active site. In particular, AHA has most of its flexibility localized on the L7 loop (β7-α7) and the C-terminal extremity of L3 (L3_C_), as well as at the L3–L5 interface, whereas PPA has the highest flexibility scattered far from the catalytic site toward the most solvent-exposed L8 and the N-terminal of L3 (L3_N_) (Figure 3 and 4).

Interestingly, in AHA mutants, flexibility (Figure 4) and correlation patterns (Figure 5) typical of wild-type AHA decrease in intensity or are even lost, whereas features typical of the dynamic signature of PPA can be detected (Figure 4 and 5). The only exception is AHA5 mutant in which more complex effects are induced on coupled motions, but not flexibility profiles, upon the introduction of the 5 mutations affecting weak intramolecular interactions. AHA mutants also present a modification of the salt-bridge interactions, their networks and distribution in clusters of spatial proximity (Figure 6 and 7). In particular, highly persistent salt bridges conserved in all the simulated variants can be detected (Figure 6 and 7; Table S1-group I in Supporting Information S1), which can be the necessary and sufficient elements, at least concerning ionic interactions, for the stabilization of the α-amylase fold. These results fit well in the context of recent MD studies demonstrating that, in the (β/α)_8_ barrel fold, coevolving residues with crucial role in function and fold stability are interconnected by intramolecular interactions, as well as they control the most important and conserved correlated and anti-correlated motions governing this fold [27]. Relevance of correlated motions in identifying clusters of critical residues for protein function and stability have also been shown in broader context and related to other protein folds [24], [51], [52]. In particular, in the group I of salt bridges (Table S1 in Supporting Information S1, Figure 6 and 7), it is relevant to mention the presence of a stable interaction between R338^A^ and the residue E19^A^ of the silent protease catalytic triad (387R^P^ and E27^P^), the latter previously proposed as a relevant residue for AHA stability and belonging to the silent protease catalytic triad of Cl^−^ - dependent α-amylases [53]. Both AHA, mutants and PPA share a salt-bridge network (D174^A^-R172^A^-D84^A^ and D197^P^-R195^P^-D96^P^) involving the catalytic nucleophile D174^A^ (D197^P^), and relying on the hub role of R172^A^ in mediating interactions both with D174^A^ and D84^A^ (Table S1 in Supporting Information S1, Figure 6 and 7). These networks could be another common elements for α-amylase fold, allowing the correct orientation of the nucleophile side chain in the catalytic site.

**Figure 8:**
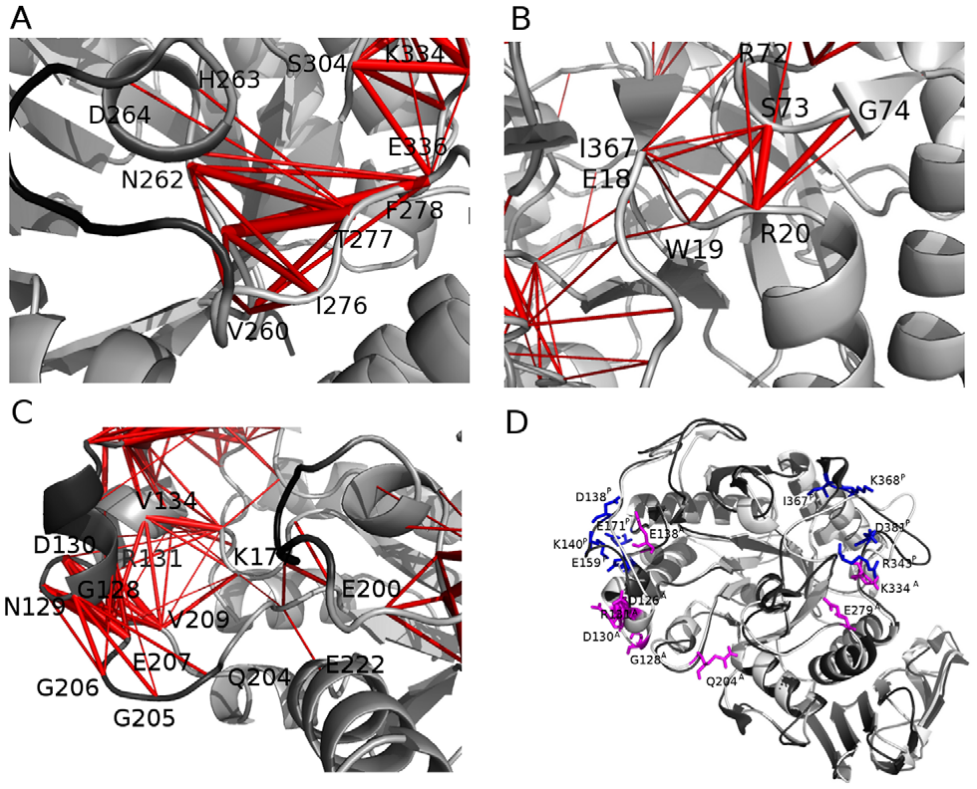
Focus on cross-correlated residues in L7–L8 region and summary of proposed residues for mutagenesis studies. A. The cross-correlation network of AHA which is lost in PPA and AHA mutants (AHA numbering) in the proximity of L7/L8 region. B. The cross-correlation network of PPA mediated by I367P not restorable in AHA mutants (PPA numbering). C. The cross-correlation network of AHA which is lost in PPA and AHA mutants (AHA numbering) in the proximity of L3/L5 loops. The positive correlations represented as red sticks connecting the Cα atoms. D. The hub residues in promoting AHA and PPA characteristic dynamic patterns, not effectively modified in the mutants and which can be a suitable residue subset to be experimentally investigated are mapped on the average 3D structures from AHA (white) and PPA (black) simulations in magenta (AHA) and blue (PPA), respectively.

A group of salt bridges (group IV, Table S1 in Supporting Information S1) and their networks, similar to that of PPA, and differing from those of AHA, can be identified in the mutants (Figure 7, 8, Table S1 in Supporting Information S1) and their relevance will be discussed in details in the next sections. This confirms a trend toward mesophilic-like properties in AHA mutants, as well as long range effects induced by the mutations. The selected mutations cannot restore the overall electrostatic networks of the mesophilic amylase, since several salt bridges of PPA lack suitable corresponding residues in AHA and the opposite (groups II and III, Table S1 in Supporting Information S1). In particular, several arginine residues of PPA are absent in AHA (Table S1 in Supporting Information S1), accounting for differential salt-bridges interactions and networks, in line with the stabilizing effects induced by replacement of AHA lysines with homoarginines [54].

### Modification in dynamics properties around L7/L8 loop

Domain A is the central and largest domain of α-amylases; it accommodates the active site at the heart of its (β/α)_8_ barrel structure. βα loops forming the substrate-binding cleft (Figure 1), have a role in substrate recognition and processing [55]–[57] in several α-amylases. In particular, loop L8 has been identified as an initial contact point for the incoming substrate, mediating enzymatic specificity [58], whereas loop L7 may rearrange upon substrate binding and release, contributing to the substrate placement inside the active site [59].

The entity of L7 flexibility is decreased in most of the mutant variants (Figure 3 and 4), accompanied by a concomitant disappearance of one of the highest correlated networks interconnecting L7 (Figure 5, Figure 8A–B) and by an emerging higher flexibility, “mesophilic-like”, in the region of L8 loop (Figure 4) in some mutant variants.

A detailed analysis of all the pairs of residues with cross-correlated motions, which are modified in AHA with respect to PPA or AHA mutants has been carried out, in particular focusing on the residues which differ in AHA and PPA, as derived by their structural alignment. In particular, a stable network of correlations connecting residues D264^A^ (which belongs to the catalytic triad), V260^A^, D261^A^, N262^A^, V275^A^, T277^A^, F278^A^ and K334^A^ in AHA (Figure 8A), is lost in the AHA mutant variants, in parallel with a local modification of the electrostatic interactions involving K334^A^. K334^A^ in AHA cooperates, by salt bridges with E336^A^ and E279^A^ and an aromatic interaction with F278^A^, to determine the correlated motions in this region and the dynamic properties of AHA L7 loop. On the contrary in PPA, only the counterparts of E336^A^ (E385^P^) and F278^A^ (F315^P^) are present, whereas K334^A^, E279^A^ are replaced by V383^P^ and W316^P^. Moreover, along with these differences, I367^P^ (which is located in the L8 insertion typical of mesophilic amylases and absent in AHA) in cooperation with the salt bridge network R343^P^-D381^P^-K368^P^ (Figure 6 and 7), plays a crucial role as critical residue in promoting a cluster of correlated motions (involving residues E10^P^, W11^P^, T71^P^, R72^P^, S73^P^, G74^P^, N75^P^) in PPA (Figure 8B) and it is related to the highest flexibility scattered toward solvent-exposed regions of L8 loop and therefore, to the different local dynamic fingerprint with respect to AHA. The correlation cluster mediated by I367^P^ and the aforementioned PPA salt bridge network cannot be established in AHA or AHA mutants since I367^P^ is located in the L8 insertion typical of mesophilic amylases (Figure S9 in Supporting Information S1) and R343^P^, D381^P^ and K368^P^ are replaced by D306^A^, N332^A^ and V318^A^, respectively. Interestingly, in the AHA mutants, a combination of local and long range effects clearly modifies the interactions and the dynamic patterns of K334^A^, favoring interactions between K334^A^ with the aforementioned D306^A^ (Figure 6 and 7) in AHA (corresponding to R343^P^ residue involved in the PPA salt bridge network), and, in turn changed the local dynamic pattern toward a “mesophilic-like” behavior. The mutations affecting K334 properties also alter the composition of the most populated cluster of electrostatic interactions typical of AHA (Figure 6 and 7; Figure S7 in Supporting Information S1). In particular, in the AHA mutants, this important salt-bridge cluster is divided into two smaller and non-communicating clusters, influencing dynamic and structural properties of the surrounding regions in the proximity of the catalytic site and including the L7/L8 regions.

It is worth to mention that the effects induced by the AHA mutations are still not completely comparable to the L8 loop flexibility pattern of PPA and its cross-correlation map. This result is not surprising considering the different length and composition of L8 loop in PPA, suggesting that a mesophilic dynamic fingerprint could be more successfully acquired combining some of the investigated AHA mutations, with other mutations or amino acid insertion typical of PPA L8 loop. In fact, integrating the data collected from the different analyses of the MD trajectories, it clearly emerged that the main determinant of differences in cross-correlated motions and flexibility in L7/L8 region in AHA mutant variants relies on a subset of residues with a pivotal role, which are suitable candidates for further mutagenesis experiments, with particular attention to the introduction of a residue corresponding to I367^P^.

However, it is striking to observe that AHA mutants manage to attain flexibility patterns similar to that of the mesophilic homolog, especially because all mutations are located far from this region, highlighting the ability of single amino-acidic changes to significantly modulate the flexibility of distal regions of AHA toward “mesophilic-like” properties.

### Modification in dynamic properties in proximity of L3 and L5 loops

Domain B is the longest unstructured region in both AHA and PPA (44 and 67 residues long, respectively) and connects β3 to α3 (Figure 1), covering one side of the central barrel and closing the substrate-binding cleft. Domain B interacts with loop β5−α5 (L5), which in part (residues 200–210) protrudes towards the active site. A hydrophobic cluster between α3, loop α3−β4, β4 and loop α4−β5 is reinforced by mutations Q164I and V196F (Figure 3), while mutation N150D allows the establishment of the D150-K190 salt bridge between α3 and α4 (Figure 7). Accordingly, mutants bearing all three these mutations (AHA5 and AHA5SS) show the lowest flexibility in the L5 region (Figure 3 and 4). V196F mutation seems to have a prominent role, since it is capable, alone, of yielding a rigidification similar to that of the multiple mutants (Figure 3 and 4).

However, the engineered mutations in AHA do not completely affect the pattern of flexibility of AHA L3 and L5 regions. In particular, the flexibility of AHA mutants is not displaced toward the L3_N_ part of the loop as in PPA (Figure 4), whereas more remarkable effects can be identified in the decreased flexibility and modification in cross-correlation patterns at the interface between L3 and L5 (observable for example in AHASS, AHAVF, AHANR and AHA5) (Figure 4 and 5). However, it is interesting to observe that the highest flexibility of AHA at the interface between L5 and L3 is strongly reduced in the mutant variants with some of them displacing the flexibility toward more exposed regions of the L5 loop. In particular, the major modifications in the dynamic properties of AHA mutants, at this site, rely on differences in the salt bridge network mediated by D203^A^ (E207^A^-K177^A^-D203^A^-K224^A^-E222^A^) (Figure 7), which is interrupted in some mutants and in PPA, (lacking D203^A^-K177^A^) as well as on the appearance in the AHA mutants of a salt bridge between residues E213^A^ and R133^A^ (whereas in PPA the corresponding glutamate residue, E249^P^, provide a salt bridge in the same area but with different orientation mediated by R158^P^), along with a general weakening of the cross-correlation networks in this region (Figure 8C). In fact, most of the AHA mutants lack or attenuate the correlated motions (Figure 8C) connecting residues in the 128–130^A^ region with residues 204–206^A^. Moreover, some AHA mutants lose correlation toward E222^A^ (belonging to the salt bridge cluster mediated by D203^A^). In the same area, PPA conserves only correlated motions involving K200^P^ (the homologous residue to K177^A^). Moreover, AHA and its mutants maintained in this regions a salt bridge cluster between D126^A^-R131^A^-D130^A^ which is absent in PPA, due to deletion and amino acidic substitutions.

In summary, the most relevant differences between AHA and PPA in 128–130^A^ and 204–206^A^ regions are Q204^A^ (L237^P^), G128^A^ (N152^P^) D126^A^ (S150^P^) and D130^A^, R131^A^ (missing in PPA). Moreover, in PPA, the displacement of flexibility toward the N-terminal portion of L3 loop is also related to the presence of two salt bridge clusters which are absent, and not allowed to be restored due to deletion or substitutions, in AHA and its mutants (D159^P^-K140^P^-E171^P^ and D138^P^-R214^P^). K106^A^ is the corresponding residue of R214^P^ but mediates in AHA and its mutants interactions with E138^A^ (Q161^P^).

In order to re-establish a PPA-like behavior in AHA mutants, a more complex network of interactions and communicating residues should be restored in this area, including pivotal residues in L3 which allow the flexibility to be displaced at the N-terminal extremity of the loop. Otherwise, mutations of AHA residues at the interface between L3 and L5 could be included to more successfully abolish the network of cross-correlated motions between the two loops, which is lacking in PPA.

### Domain C: a structural domain shielding hydrophobic residues of the catalytic domain

Domain C is composed of 8 β strands arranged in a greek-key motif and tightly packed against α6, α7 and α 8 of the barrel (Figure 1). The interface between domains C and A is rich in aromatic and hydrophobic residues which contribute to the stabilization of the interdomain interface, as well as they mediate the extension of the hydrophobic core of domain A towards domain C [33]. The sandwich-like structure of domain C features, in both AHA and PPA, asymmetry between the two composing β-sheets: the buried β-sheet constitutes the interface with domain A and is stable and ordered, while the solvent-exposed β-sheet is highly disordered, and split into 2 separate 2-stranded β-sheets (Figure S9 in Supporting Information S1). The latter ones are highly persistent in PPA, whereas in AHA main chain H-bonds stabilizing the central part of these sheets are weakened. Interestingly, most of the mutants display PPA-like H-bond pattern (with the exception of AHA5SS) and the presence of a PPA-like salt bridge involving the C-terminal residues of the proteins, along with disappearance of some salt bridges at the interface between domain A and C typical of AHA (Figure 7; Table S1 in Supporting Information S1). This behaviour was unexpected. In fact, domain C does not carry any of the studied mutations, and nevertheless, shows structural and dynamical differences in mutated forms, demonstrating that single mutations have global and long range effects, affecting regions very distant from the site of mutation.

## Discussion

Intramolecular weak interactions have a fundamental role in stabilizing protein structures. Special attention has been given to their role in the context of thermal adaptation of proteins [7], [44], [60], with particular regard to electrostatic interactions [46], [61]–[64]. The present study provides molecular details related to the observed variations in thermal stability and kinetic parameters of AHA mutants with respect to the *wild-type* cold- and warm-adapted counterparts, as well as it points out long range effects induced by the mutations.

Our MD investigation shows that the AHA mutations are capable of eliciting effects, in agreement with the restoring or strengthening of the target interactions, on the dynamic environment of the mutated residues in a mesophilic-like direction. If the minor entity of the introduced mutations is considered, since few residues in a multi-domain protein of about 500 amino acids are mutated, it is striking to observe the wide range of different dynamical behaviours these mutants exhibit.

Interestingly, the local effects, in the mutation sites, are also able to modify the dynamic character of the mutants, producing complex distal effects that underlie the intimate interplay between hydrophobicity, electrostatic interactions and protein dynamics.

The mutations are extremely effective in modifying AHA structural and flexibility properties, as hypothesized by the experimental characterization [37], showing also that, in the case of multiple mutants, the modification of activity and thermal stability to mesophilic values is strongly advantaged. The ability of AHA mutations to elicit a mesophilic-like behaviour is confirmed in the results of MD simulations, where the comparison is restricted exclusively to structural and dynamic properties. In fact, the mutants exhibit secondary structure persistence, flexibility, correlated motions, electrostatic interactions, and, hydrophobicity in-between of those seen for the two wild-type enzymes.

However, fundamental differences both in the structure and the sequence of the two wild-type enzymes still exist and make it impossible for these mutations alone to reproduce the full set of stabilizing local interactions seen in PPA. In fact, our investigation also shown, in excellent agreement with the experimental values, that the conversion of AHA in a mesophilic enzyme cannot be completely achieved by the introduction of these mutations alone. In fact, other substitutions, highlighted in our study, should be considered to get more efficient modification of AHA toward the mesophilic counterpart, as summarized in Figure 8D, as K334^A^ or I367^P^.

Our MD simulations of AHA mutant variants and their comparison to dynamic features of the wild-type counterparts also point out other long range effects induced by the mutations. In particular, the effect of flexibility is largely apparent in regions featuring high flexibility in AHA (loop 7) and PPA (loop 8), which mainly cluster in regions near the active site and substrate-binding groove, and long range effects are even transmitted to the C-terminal domain. In this context, the present investigation provides atomic-detailed evidence of the ability of the selected mutations to modulate the dynamic properties of AHA, and offers unprecedented insight in the way this modulation takes place in a large protein. Interestingly, a correlation (with a Pearson correlation coefficient higher than 0.78) exists between the combination of mesophilic-like salt bridge interactions acquired by the AHA mutants (highlighted in Table S1 in Supporting Information S1) and the experimentally determined k_cat_/K_m_ and Tm values of each mutant [37]. These interactions include, in particular, the restored PPA-like D150^A^-K190^A^ salt-bridge, the loss of K177^A^-D203^A^ salt-bridge and the modification of interactions involving K334^A^.

In a broader context, the approach applied here, integrating different properties related to protein dynamics (in particular the parallel and integrated evaluation of correlated motions and electrostatic interaction network), could be useful to predict and rationalize the effects induced by a mutation on protein activity and stability of an extremophilic enzymes, if comparative studies with other temperature-adapted counterparts can be carried out.

## Materials and Methods

### System setup and molecular dynamics simulations

The X-ray structure of AHA (PDB entry 1AQH [33]) was *in silico* modified to model 7 mutant forms: 5 single mutants AHANR (N12R), AHAQI (Q164I), AHAVF (V196F) and AHATV (T232V) [35]; one double mutant AHASS (Q58C A99C) and 2 multiple mutants AHA5 (N150D, Q164I, V196F, T232V, K300R) and AHA5SS (Q58C, A99C, N150D, Q164I, V196F, T232V, K300R) [37]. MD simulations were performed using GROMOS96 force-field with the GROMACS software (www.gromacs.org). The mutated forms of AHA were soaked in a dodecahedral box including counterions (Na^+^) and SPC [65] water molecules using periodic boundary conditions, with a minimum distance between the solute and the box of 0.5 nm (average box size of 527.66 nm^3^ including 49893 atoms) and the systems were equilibrated in several steps [50]. Productive MD simulations were performed in the NPT ensemble (300K, 1 bar and 2 fs time-step with LINCS algorithm [66]). Electrostatic interactions were calculated using PME [67] summation scheme. Van der Waals and Coulomb interactions were truncated at 0.8 nm and conformations stored every 2 ps. To improve conformational sampling, six independent 6 ns simulations were carried out for each system. MD simulations showing the slowest convergence of root mean square deviation (rmsd) values have been elongated up to 10 ns (*run* 2 of AHAQI and *run 3* of AHA5SS) and the first 0.2–2 ns depending on the system have been discarded to assure stability of the trajectories. In fact, MD multiple-*replica* approach allows a wider conformational sampling than only few longer molecular dynamics simulations, especially in the case of large multi-domain proteins as AHA is, if the stability of the trajectory and convergence of the analyzed properties have been carefully verified [68]–[71]. The equilibrated portions of the simulations of the same system were joined into a macro-trajectory. The conformational *ensemble* collected for each mutant is comparable, in terms of simulations protocol, degree of essential motions captured by the first principal components and number of frames in the collected ensemble to that achieved for the wild-type cold- (AHA) and warm-adapted (PPA) adapted amylases previously published [50], in order to carry out a prompt comparison of dynamical properties.

### Molecular and structural properties

Secondary structure was determined for all stored conformations using DSSP [72] and the most frequently attained secondary structure for each residue was evaluated to obtain a residue-dependent persistence degree of secondary structure profile (PDSSP). Intramolecular hydrogen bonds (H-bonds) were evaluated using both a donor-acceptor distance cut-off of 0.35 nm and an acceptor-donor-hydrogen angular cut-off (≤30°); *ensemble* averages were normalized using the total number of donors and acceptors.

Surrounding hydrophobicity (Hp) was computed for each residue according to [73]. In particular Hp was computed for each residue as the sum of hydrophobicity indexes, obtained from thermodynamic transfer experiments [74], of the surrounding residues within a 0.8 nm distance cutoff:
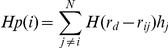
where the summation runs over the whole protein, *r_ij_* is the distance between the Cα atoms of residues *i* and *j*, *H(x)* is the Heaviside step function [H(x) = 1 if x≥0, and zero otherwise], *r_d_* is the distance cutoff and *h_j_* is the hydrophobicity index of residues *j* in kcal/mol. The adopted cutoff has been demonstrated to be suitable to characterize the hydrophobic behaviour of amino acids and to accommodate both the local and non local interactions [73], [75].

Hp is therefore a measure of the tendency of the environment of each residue to exclude water, computed on the basis of the 3D structure using experimental information. Computed on equilibrium ensembles obtained from MD simulations, it gives an indication of the relative strength and stability of the hydrophobic clusters in a protein. It should be in principle able of identifying the effects of a mutation taking into account both the short-range effects, due to changes in the physico-chemical properties of the mutated residues, and the long-range effects of mutation on structure and dynamics. However, it is worth mentioning that the accuracy of the computed values is hindered, independently of the hydrophobicity scale adopted, by two strong assumptions. In fact, by summing up the contributions of different residues, the Hp calculation assumes that these contributions are independent and therefore additive, which may lead to misleading conclusions. On the other hand, the use of hydrophobicity indexes assumes that these values are transferable from the experimental study to the analysis of folded protein structures [76].

Residue-based comparison of the proteins was carried out according to a manually-checked structural alignment with DALI (Figure S8 in Supporting Information S1). Domains A, B and C of AHA are composed of residues 1–86/147–356, 87–146 and 357–448, respectively, whereas domains A, B and C of PPA are composed of residues 1–98/170–404, 99–169 and 405–496. Amino acid numbers in the text are labelled with a superscript A or P, referring to the AHA or PPA sequences, respectively. The 8 β-strands and the 8 α-helices, composing the (β-α)-barrel of domain A, are referred to as β1 to β8 and α1 to α8 according to their order in the sequence.

### Protein dynamics

The essential dynamics (ED) analysis reveals high-amplitude concerted motions in the equilibrated portion of the trajectories, based on the diagonalization of the Cα covariance matrix of the atomic positional fluctuations [77]. About 10 eigenvectors are required to obtain the description of 70% of the variance in the simulated proteins. The collection of the selected eigenvectors describing the collective motions is termed essential subspace and can describe protein motions at a reasonable level of accuracy. Motions described by the essential subspace have been mapped on average 3D-structures by representing with ellipsoids (at probability 0.1) the computed anisotropic temperature factors obtained from the per-residue Cα anisotropic U-tensors of cross-correlations of motion. The average 3D-structure is defined as the sampled conformation with the least distance (in terms of main-chain rmsd) from the *ensemble* average of atomic positions. The cosine content and overlap indexes as root mean square inner product have been monitored to evaluate the sampling quality, according to standard procedures applied in our laboratory [78].

The per-residue Cα root mean square fluctuation (rmsf) was calculated with respect to the average structure, after projection of the trajectories on the essential subspace. To properly assess the rmsf convergence and the consistency of flexibility profiles, per-residue rmsf profiles have been computed on partially overlapping time-windows, starting from the first frame of the macro-trajectories and moving onwards by steps of 500 ps. This calculation has been repeated for different window lengths (1, 2, 5, 10, 15 and 20 ns) and an average rmsf profile for each time-window has been calculated (Figure S10 in Supporting Information S1).

Correlation plots were obtained by first computing Cα correlation matrices [26] *C(i,j)*, using non-overlapping averaging windows of 1 ns (Figure S11 in Supporting Information S1), and also compared, for validation, to correlations on averaging windows of 2 and 3 ns. *C(i,j)* has been calculated according to,
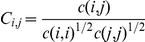
Where *c(i,j)* is the covariance matrix of protein fluctuations between residues *i* and *j*.

Only the most significant (|*C(i,j)*|>0.4) long range (|*i−j*|>12) correlations have been considered. The cutoff of sequence distance has been selected to exclude from the analysis correlations relative to α-helices structure and contiguous residues in the primary sequence. Moreover, since an average *C(i,j)* matrix has been considered for the analysis (even if the single *C(i,j)* matrix relative to each 1 ns window have been compared to the average one), a cutoff of 0.4 in absolute value have been selected to identify significant correlations and to exclude pairs of residues which are poorly communicating each other and likely to be characterized by uncoupled motions. In particular, on 1 ns time-scale, only positively correlated motions are identified above this cutoff. Moreover, in order to clearly identify differences in the patterns of coupled motions of AHA and PPA functional regions, in the correlation plots the correlations relative to secondary structural elements (correlations within the β-barrel and correlations between α-helices) have been discarded. Correlations were then plotted on the 3D structures by connecting atoms *i* and *j* with lines, with thickness proportional to *C(i,j)*.

### Salt bridge interactions and networks

Salt bridges were identified as oppositely charged groups at less than 0.4 nm of distance in at least 24% of the macro-trajectory frames. In order to check the validity of the results also other distance (0.45 and 0.5 nm) and persistence (20%) cutoffs were employed (*data not shown*). The cut-off of 24%, in particular, was selected as the persistence value which best divided the interactions dataset in well-separated groups, defined as signal and noise (Figure S12 in Supporting Information S1), according to a protocol previously applied [48] and summarized in Figure S12 in the Supporting Information S1. In particular, the distribution of charge-charge pairs at a defined cutoff of distance has been analyzed in terms of probability density function. It turns out that there are several ion pairs at low persistence (<10%) which are likely to be not relevant for protein structure and dynamics and identified as “noise” signal. Whereas, at persistence greater of 30% the number of ionic pairs is generally constant. Therefore, the selected cutoff of 24% should be able to divide the dataset in the two regions of low and high significance. This cutoff has been calculated by two supervised classification methods, trained on a set composed by two classes: noise (interactions below 10%) and signal (interactions above 30%). A Support Vector Machine (SVM) and a k-Nearest Neighbours (kNN, k = 4) classifiers, as implemented in Matlab suite, have been trained on this set and used to classify all the interactions between 10 and 30%.

To identify the clusters of spatial proximity of salt-bridges, the residues involved in salt bridges have been represented as nodes of an unrooted unoriented graph, in which two nodes were connected by arcs if an interaction was found between them or if two of them were at less than 5 residues of distance in the sequence. An exhaustive search procedure was carried out on the graph to isolate the networks of interactions, which have been represented as interaction clusters (indicated by different colors in Figure 6 and 7).

## Supporting Information

Supporting Information S1 **This file contains all the supporting tables and figures for this article.**
(PDF)
