# Supplementary material for: Molecular Dynamics of Mesophilic-Like Mutants of a Cold-Adapted Enzyme: Insights into Distal Effects Induced by the Mutations
Source: PLoS One. 2011 Sep 7;6(9):e24214. doi: 10.1371/journal.pone.0024214 (PMC3168468; doi:10.1371/journal.pone.0024214)

Table S1. Salt-bridge persistence. *I.* Indicates salt-bridges common to all the simulated variants; *II.* Lists of salt-bridges which are typical of PPA and cannot be restored in AHA mutants due to amino acidic substitutions in AHA; *III.* List of salt-bridges in AHA variants and lacking in PPA due to amino acidic substitutions; *IV.* Indicates salt-bridges which change from AHA to AHA mutants variants in a "mesophilic-like" direction. Arginine residues of PPA and absent in AHA, which account for differential salt-bridge interactions in the two proteins are highlighted in grey. The most relevant salt-bridge clusters involved in determining mesophilic-like properties are highlighted in blue and have a Pearson correlation coefficient of about 0.78 with the experimental parameters.

aa^{AHA}	AHA	AHASS	AHAVF	AHANR	AHA5SS	AHAQI	AHATV	AHA5	PPA	aa^{PPA}
I.salt-bridges common to AHA, mutants and PPA										
**E10-R49**	85.98	93.03	78.58	94.71	56.28	88.19	82.88	83.61	99.35	**E18-R61**
**E10-R60**	65.75	79.30	57.63	65.03	67.17	77.87	58.23	71.41	92.95	**E18-R72**
**E19-R338**	99.85	99.89	99.87	99.97	100	99.95	99.48	100	86.83	**E27-R387**
**E21-R73**	96.74	96.21	93.94	96.35	97.71	94.43	93.29	91.97	88.70	**E29-R85**
**R64-D162**	56.15	60.96	42.97	60.84	52.73	59.09	60.60	65.93	26.67	**E76-K185**
**D69-R73**	58.37	60.99	58.57	52.60	48.93	64.59	67.08	58.85	72.43	**D81-R85**
**D84-R172**	97.50	98.53	99.96	99.14	99.99	99.90	99.84	98.94	99.97	**D96-R195**
**R172-D174**	99.79	98.27	80.83	90.70	99.82	96.18	93.34	65.85	96.96	**R195-D197**
**K177-E207**	83.13	93.45	96.91	73.32	86.75	77.51	87.83	75.55	60.48	**K200-E240**
**D203-K224**	68.62	78.29	83.44	80.91	89.50	73.03	67.76	75.62	38.21	**D236-K257**
**E222-K224**	66.38	88.62	78.98	86.91	96.93	79.66	53.62	73.72	77.53	**E255-K257**
**R234-D280**	43.24	72.69	52.04	84.10	58.19	50.72	80.07	82.98	48.17	**R267-D317**
II. salt-bridges lacking in AHA and AHA mutants										
**N12-P319**	--	--	--	--	--	--	--	--	58.28	**R20-E369**
**E19-Q22**	--	--	--	--	--	--	--	--	98.24	**E27-R30**
**E39-E56**	--	--	--	--	--	--	--	--	50.85	**E47-K68**
**S44-48T**	--	--	--	--	--	--	--	--	65.05	**R56-E60**
**T48-R60**	--	--	--	--	--	--	--	--	90.51	**E60-R72**
**R64-68I**	--	--	--	--	--	--	--	--	64.05	**E76-R80**
**K106-E118**	--	--	--	--	--	--	--	--	75.94	**R124-D138**
**D115-S149**	--	--	--	--	--	--	--	--	45.71	**D135-K172**
**Gap-N136**	--	--	--	--	--	--	--	--	78.95	**K140-D159**
**Gap-A148**	--	--	--	--	--	--	--	--	75.55	**K140-E171**
**Q135-E213**	--	--	--	--	--	--	--	--	76.91	**R158-E246**
**N150-Q153**	--	--	--	--	--	--	--	--	44.07	**D173-R176**
**A158-D162**	--	--	--	--	--	--	--	--	64.48	**D181-K185**
**A189-S194**	--	--	--	--	--	--	--	--	27.06	**D212-R227**
**E228-G236**	--	--	--	--	--	--	--	--	48.08	**K261-E272**
**N235-E384**	--	--	--	--	--	--	--	--	55.31	**K268-D432**
**N235-D385**	--	--	--	--	--	--	--	--	27.92	**K268-D433**
**S242-D363**	--	--	--	--	--	--	--	--	81.59	**K278-D411**
**S254-S255**	--	--	--	--	--	--	--	--	43.03	**D290-R291**
**D285-Y341**	--	--	--	--	--	--	--	--	99.11	**K322-E390**
**G309-gap**	--	--	--	--	--	--	--	--	81.87	**R346-D353**
**V318-N332**	--	--	--	--	--	--	--	--	38.04	**K368-D381**
**S340-Y341**	--	--	--	--	--	--	--	--	60.07	**R389-E390**
**S340-W436**	--	--	--	--	--	--	--	--	51.05	**R389-D485**
**A353-R373**	--	--	--	--	--	--	--	--	26.26	**D402-R421**
III. Salt-bridges lacking in PPA										
**K27-D347**	77.75	44.19	46.50	77.49	49.06	30.89	47.07	63.01	--	**K35-W396**
**D80-K169**	26.87	57.20	73.88	36.55	32.34	29.94	61.21	58.63	--	**R92-A192**

**K106-E138**	85.65	71.93	79.17	79.67	84.85	80.40	89.89	80.95	--	**R124-Q161**
**D126-R133**	55.53	79.01	72.49	71.63	61.44	71.53	82.19	58.14	--	**S150-Q156**
**D130-R133**	44.18	48.27	28.32	33.77	33.34	37.49	42.14	35.22	--	**Gap-Q156**
**R282-D285**	34.35	74.92	61.09	56.05	39.28	34.54	56.18	61.75	--	**R319-K322**
**R282-D437**	46.95	81.04	80.32	68.31	46.35	52.25	67.83	71.21	--	**R319-P486**
**D354-K443**	72.39	62.22	64.97	85.66	68.89	70.05	76.90	53.90	--	**G403-A492**
**K406-E419**	68.33	59.89	52.17	63.79	63.71	57.95	77.16	64.06	--	**S454-I465**
**K406-D437**	87.24	79.58	70.71	80.71	73.17	76.65	84.69	75.39	--	**S454-P486**
IV. Salt-bridges "mesophilic-like" in AHA mutants										
**R12-D15**	--	--	--	63.09	--	--	--	--	97.16	**R20-D23**
**E56-R64**	24.44	--	35.28	--	--	--	--	32.14	--	**K68-E76**
**N150-K190**	--	--	--	--	58.17	--	--	66.97	30.22	**D173-K213**
**K177-D203**	46.43	51.98	72.58	33.45	--	--	80.23	24.25	--	**K200-D236**
**E279-K334**	69.60	--	--	--	--	--	--	--	--	**W316-383V**
**D306-K334***	--	33.96	76.23	54.17	--	60.51	36.48	39.71	28.83	**R343-D381**
**K334-E336**	94.69	47.91	77.88	51.62	62.97	25.39	75.77	43.10	--	**V383-E385**
**K414-Cterm**	--	36.16	43.26	--	34.31	33.18	34.65	--	--	**N460-Cterm**
**K446-Cterm**	--	--	--	35.90	--	--	--	42.33	36.62	**K495-Cterm**

Figure S1. Kinetic (k_{cat} and K_m and k_{cat}/K_m) and thermodynamic (T_m) parameters are shown, as reported in refs. (29, 31 in the Main Text).

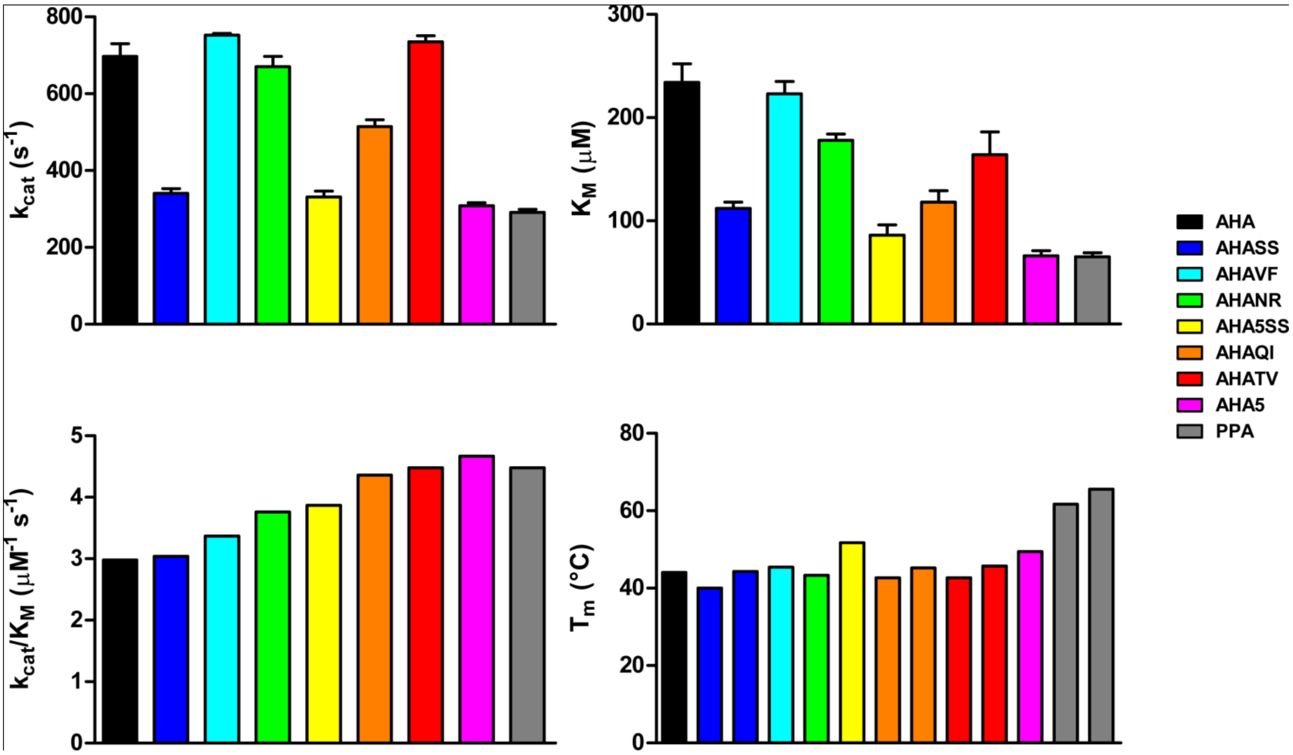

Figure S2. Localization on the 3D structure of N12R mutation. Secondary structural elements are shown as cartoon and residues involved in salt-bridges networks in are indicated in black and green in PPA and AHANR, respectively. The residues $R12^{A}/R20^{P}$ and the residue $D15^{A}/D23^{P}$ are shown in orange and cyan respectively.

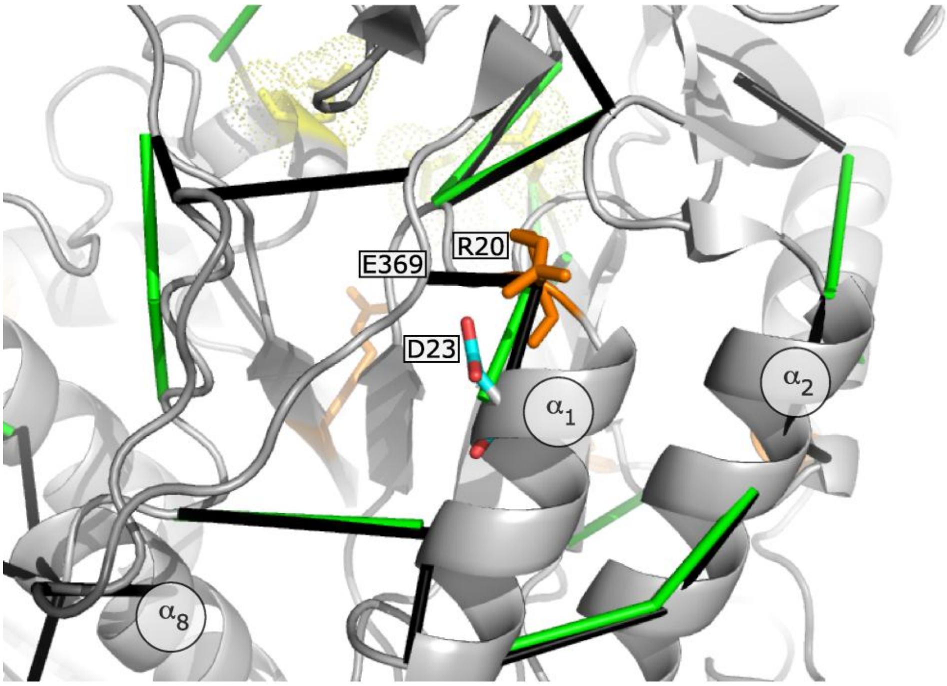

Figure S3. Surroundings of AHA mutant residues engineered to restore salt-bridge or aromatic interactions typical of PPA. (A) Persistence degree of the residues in the surrounding (<6 Å) of N12R mutation of AHA and its corresponding residue in PPA (R20^{P}). (B) Localization on the 3D structure of PPA of D173^{P} (orange), which corresponds to the N150D mutation in AHA, and its salt-bridge network in PPA (black lines). (C) Persistence degree of the residues in the surrounding (<6 Å) of Q164I mutation of AHA and its corresponding residue in PPA (I187^{P}). (D) Localization on the 3D structure of I164F mutation in AHA (orange stick) and hydrophobic residues observed with a cut-off of 8Å (light blue sticks).

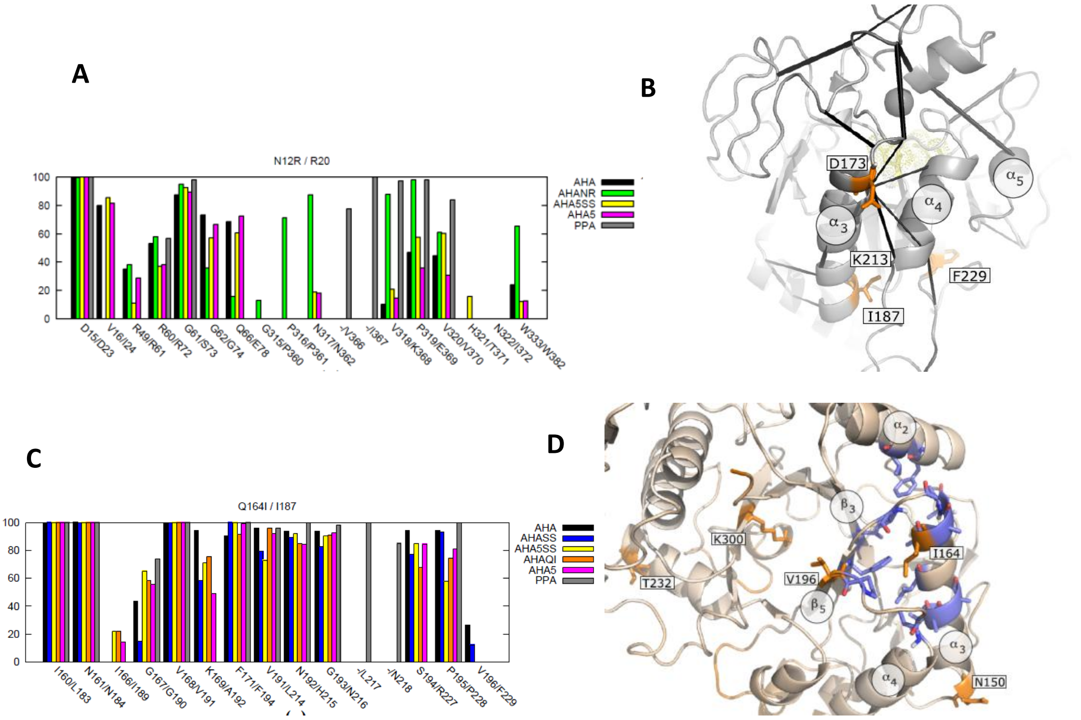

Figure S4. **Localization on the 3D structure of V196F mutation.** Secondary structural elements are shown as cartoon. The residues V196[A], K300[A] and Q164[A], which have been replaced by PPA residues in the different mutant variants and are in proximity each other, are indicated as orange sticks. The aromatic residues which can interact with F196[A] in V196F mutants, conserved both in AHA and PPA, are shown in green (Y82[A] and F198[A]).

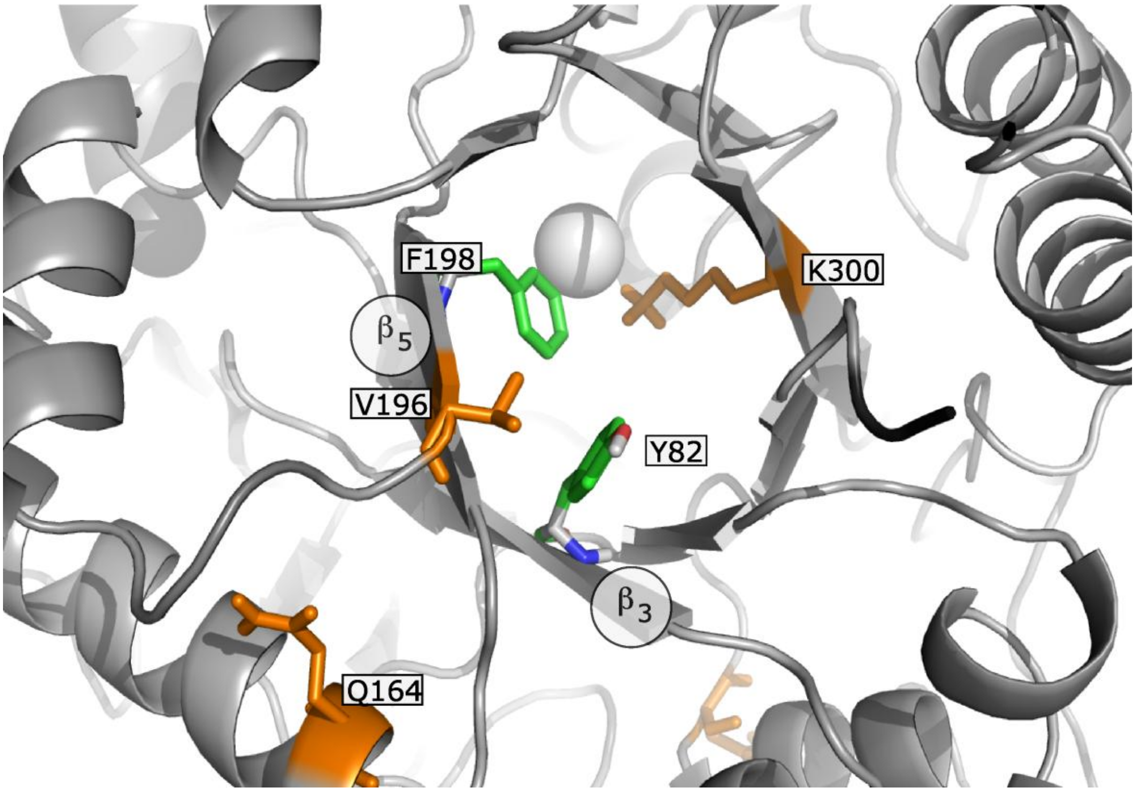

Figure S5. Surroundings of V196F and T232V mutations. (A) Persistence degree of the residues in the surroundings (6 Å) of V196F mutation of AHA and its corresponding residue in PPA (F229[P]). (B) Persistence degree of the residues in the surroundings of T232V mutation of AHA and its corresponding residue in PPA (V265[P]) (6 Å). (C) Localization on the 3D structure of T232V mutation in AHA (orange stick) and hydrophobic residues observed with a cut-off of 6Å (light blue sticks). (D) Persistence degree of the residues in the surroundings of K300R mutation of AHA and its corresponding residue in PPA (R337[P]).

A

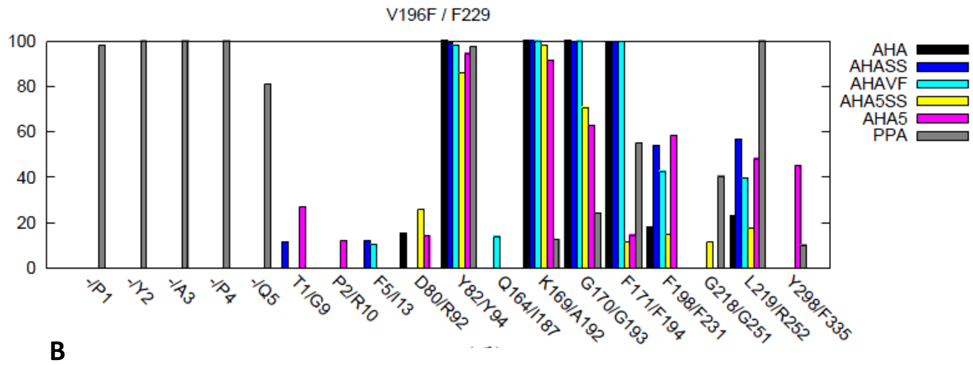

B

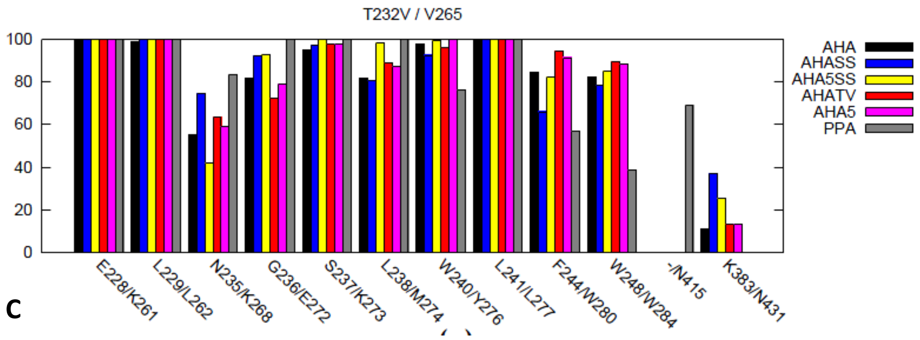

C

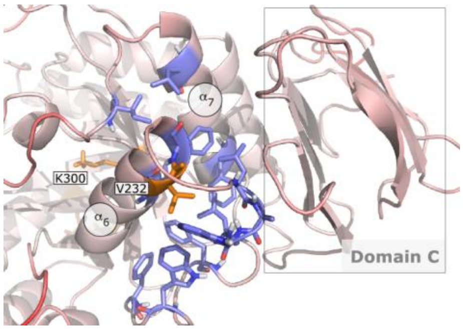

D

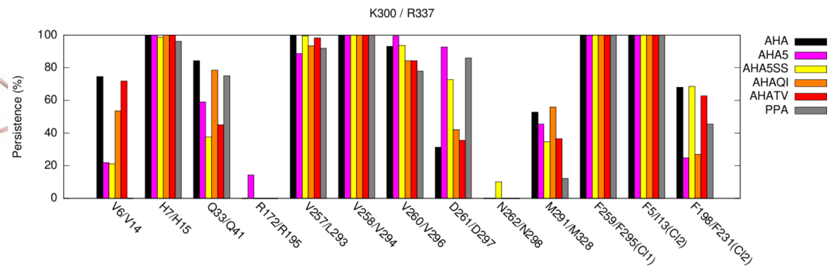

Figure S6. The 3 macro-clusters of aromatic interactions in the surroundings of the 300Lys^{A}/337Arg^{P} mapped on the 3D structure of AHA.

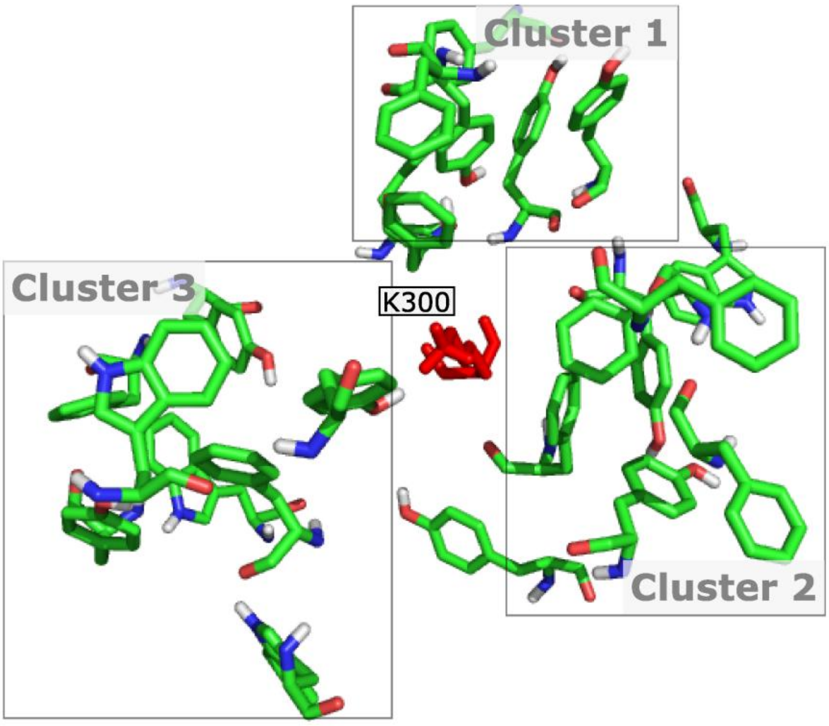

Figure S7. Most populated cluster of salt-bridges in AHA (cluster 1) in comparison to salt-bridge clusters of mutant variants. For sake of clarity AHASS is reported as representative of the AHA mutants. Mutations induce modifications in the interactions and correlations mediated by K334, which cause a different composition of the main salt-bridges clusters between the wild-type cold-adapted enzyme and its mutant. In particular, in the mutants, the AHA cluster 1 of salt-bridges in divided into two smaller clusters (bottom-right and bottom-left panels), namely cluster1a and cluster1b, altering dynamic and structural properties of regions in the proximity of the catalytic site.

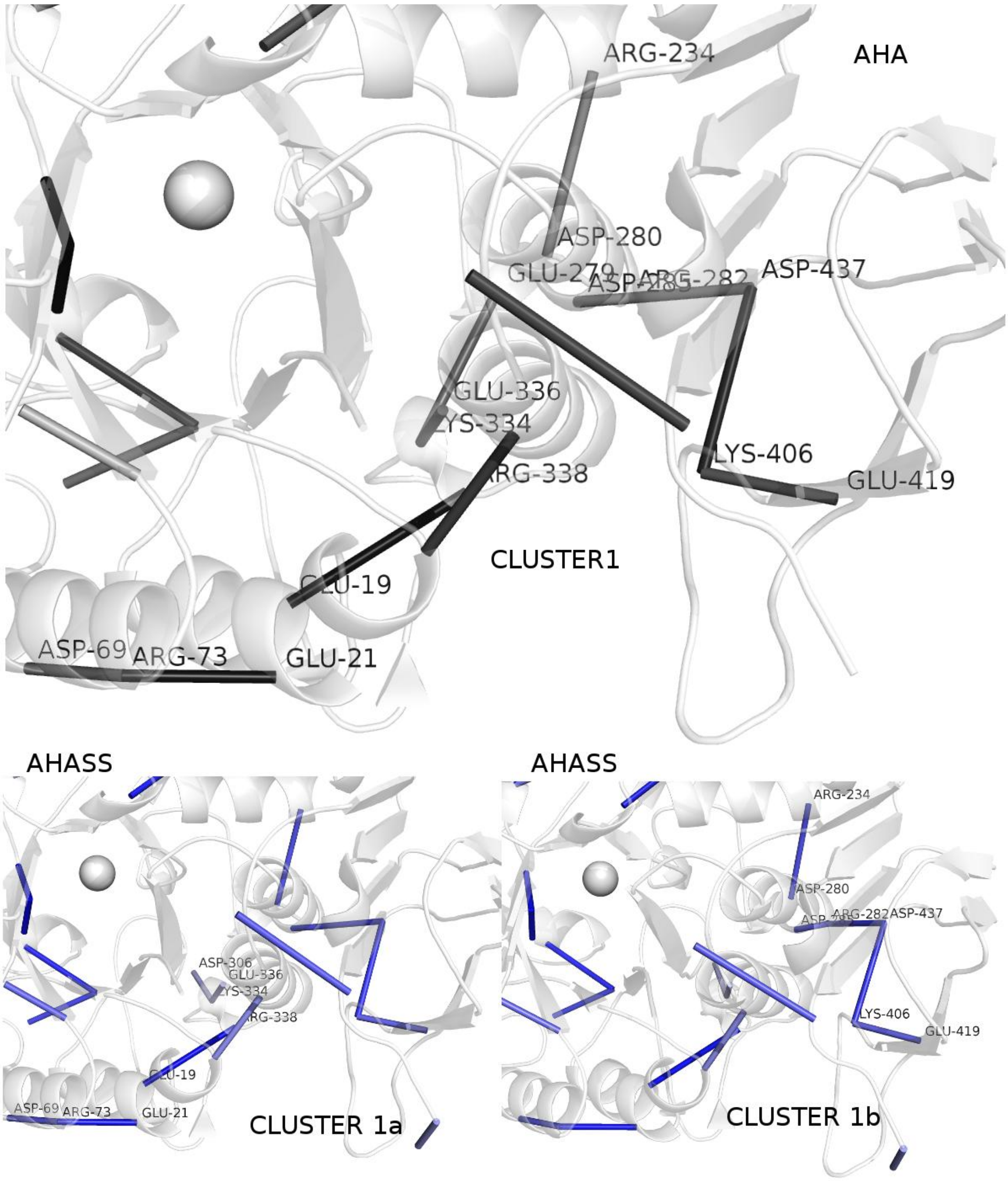

Figure S8. Structural alignment of AHA and PPA. Structural alignment between AHA and PPA, obtained with DaliLite [Holm L and Park J, Bioinformatics, 2000]. Secondary structures for the two enzymes have been calculated using DSSP on their crystal structures (PDB ids 1AQH and 1PIF, respectively). The alignment has been formatted using ALINE [Bond CS and Schuettelkopf AW, Biological crystallography, 2009].

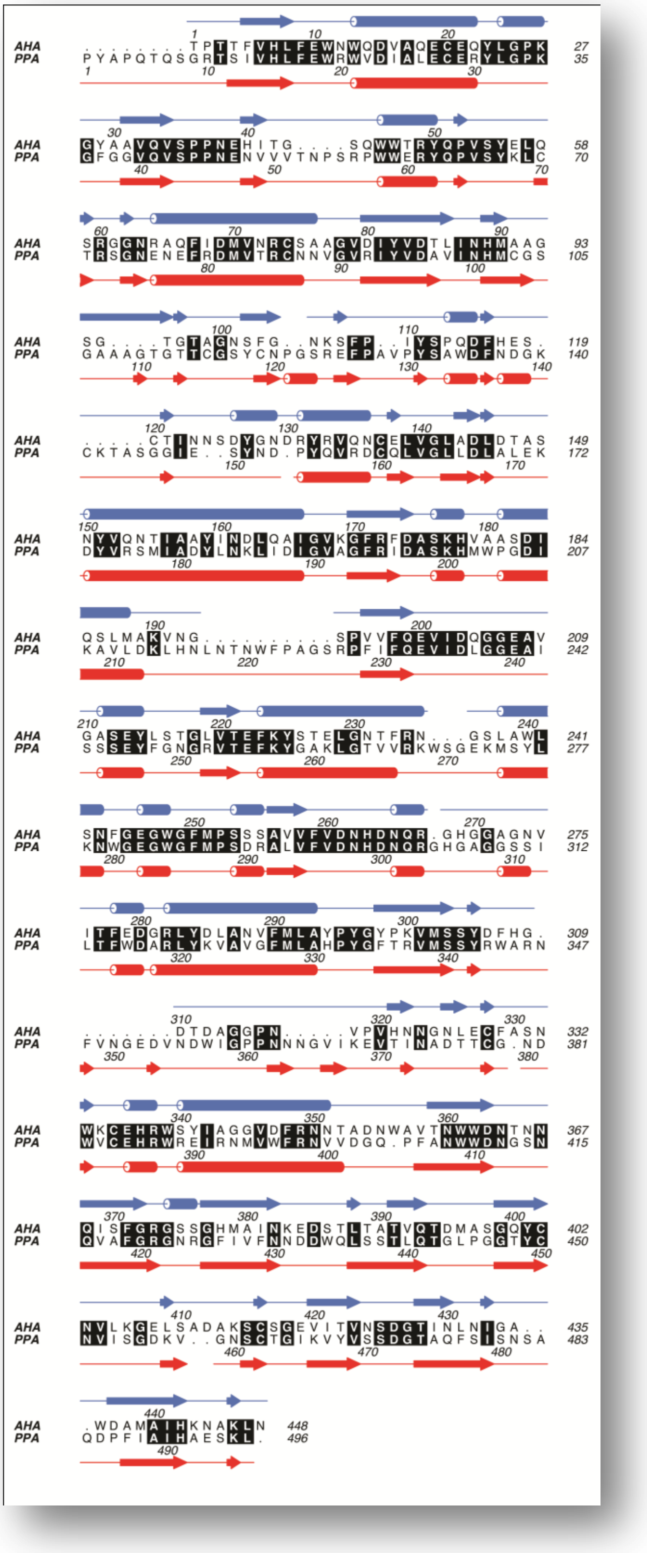

Figure S9. Rmsf and Hp profiles of domain C in AHA, PPA and AHA mutants. Secondary structure of domain C in AHA (a) and PPA (b); the two strands described in the text are highlighted in red to distinguish them from the β-strands of the domain A barrel. (c) Rmsf and Hp profiles of domain C.

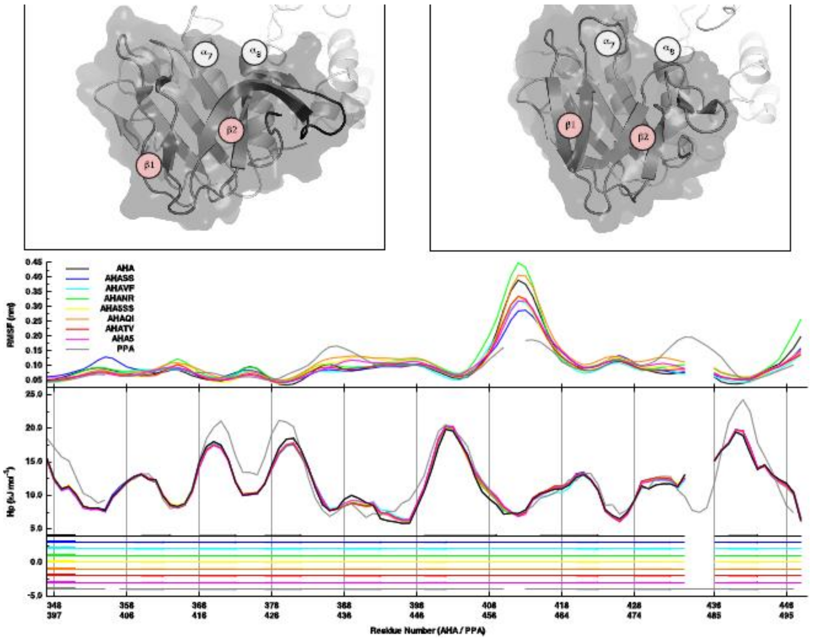

Figure S10. Rmsf profiles calculated on partially overlapping windows of different time lengths. The flexibility profile computed on the 20 ns time-windows well reproduced both quantitatively and qualitatively the ones calculated on the full 31 ns meta-trajectories, thus showing that the calculated property has reached a reasonable convergence level. AHAQI case is reported as an example.

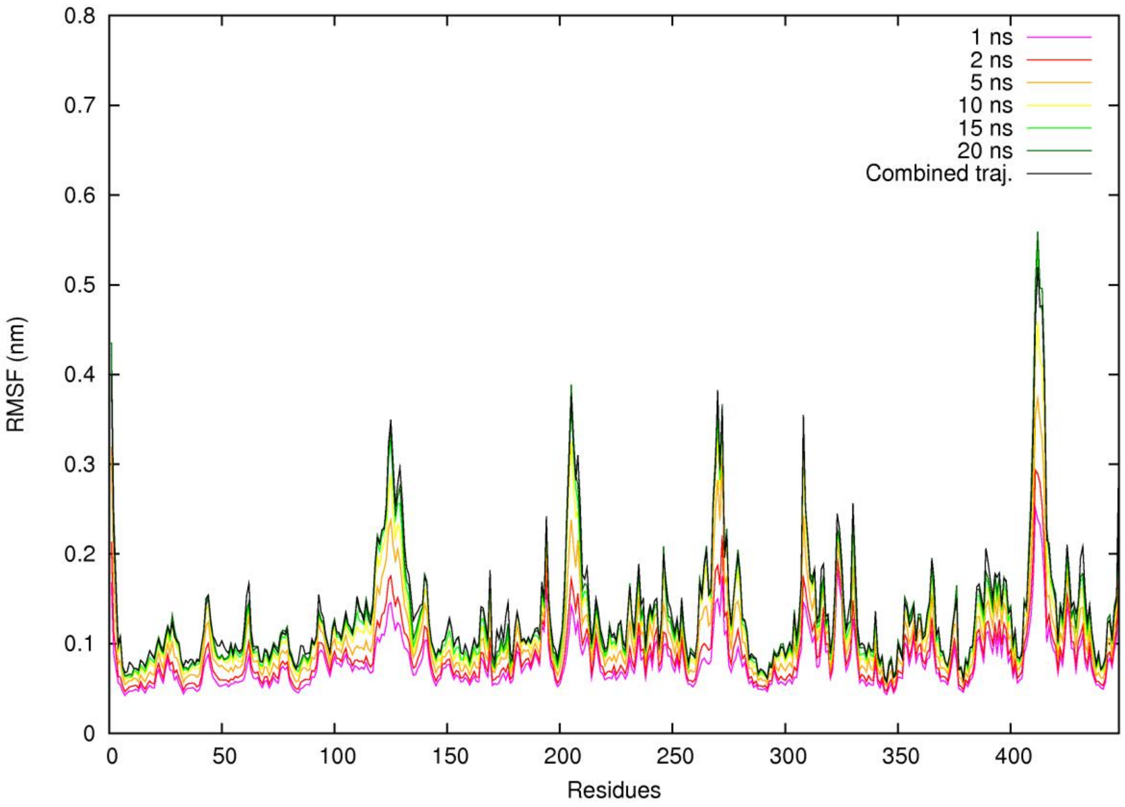

Figure S11. Dynamical Cross-Correlation Matrices (DCCM) of AHA, AHA mutants and PPA, calculated as averages from non-overlapping windows of 1 ns. Positive correlations are in the upper left triangle, negative correlations in the lower right triangle.

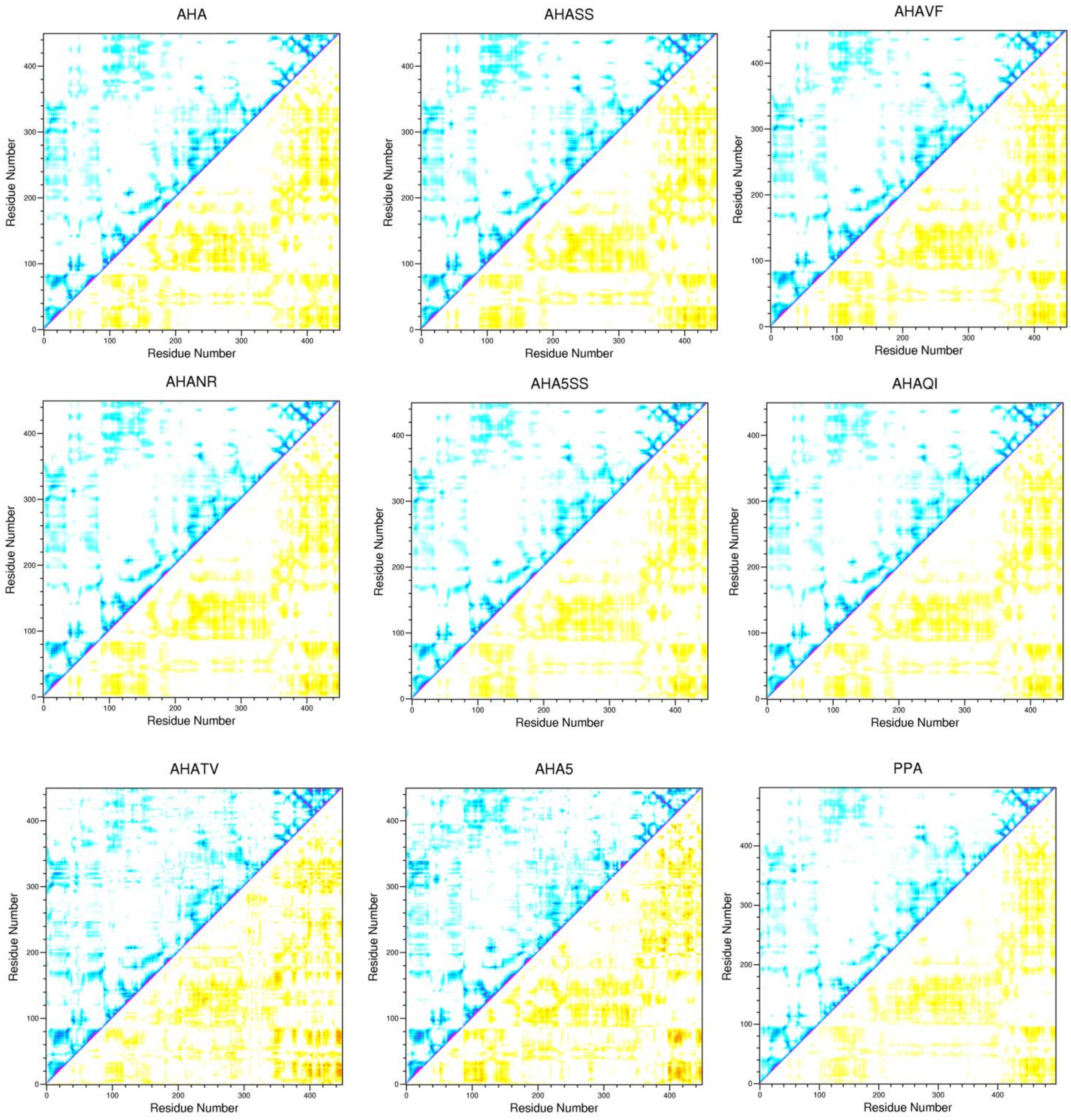

Figure S12. Determination of significance cut-off of salt-bridges persistence. A) Charge-charge interactions ranging from 0.01% to 100% of persistence from AHA and PPA, as explained in Methods, were used to obtain an estimation of the probability density function, which describes the relative likelihood of finding an interaction for a given persistence value. This was performed by employing the kernel density estimation algorithm as implemented in the Matlab suite. B) Each electrostatic interaction from AHA and PPA was initially considered when two oppositely charged groups were found at less than 0.4 nm in at least the 0.01% of the combined trajectory frames. Trajectories of AHA mutants were not considered, as including their interaction would have resulted in biasing the data set for AHA-like salt bridges. Each selected interaction is shown as a histogram box in the plot. Interactions below 10% and above 30% of persistence were considered, respectively, as the noise and the signal classes, and used as the training set for a Support Vector Machine (SVM) and a k-Nearest Neighbors (kNN, k=4) classifier, as implemented in the Matlab suite. The trained classifiers were then used to classify all the interactions between 10 and 30% of persistence. Both algorithms divided this set between 20 and 24%. 24% was thus chosen as the persistence cut-off (black horizontal line in the plot).

A

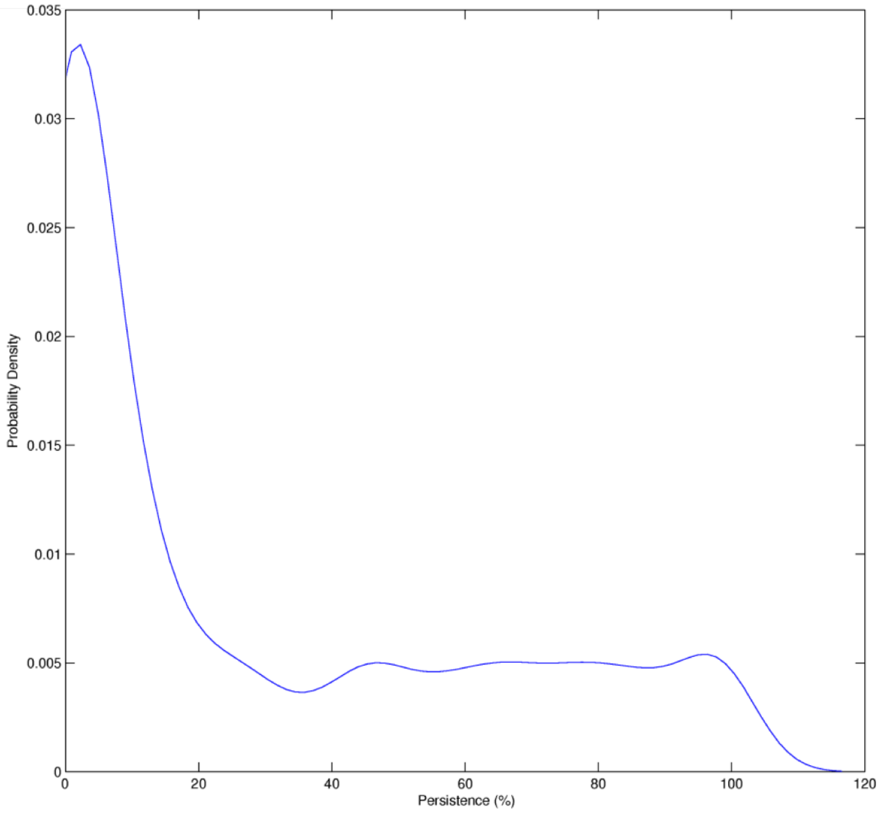

B

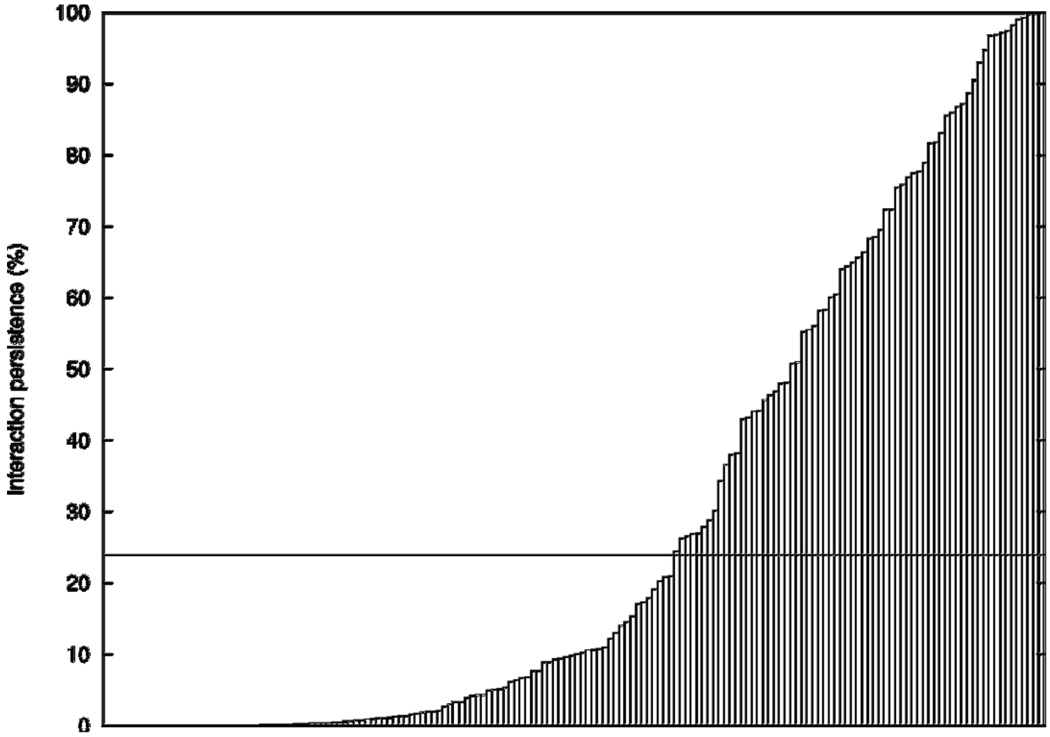

Supplement: Supporting Information S1 — This file contains all the supporting tables and figures for this article. (PDF) [file pone.0024214.s001.pdf]
